# Cystic fibrosis carriership and tuberculosis: hints toward an evolutionary selective advantage based on data from the Brazilian territory

**DOI:** 10.1186/s12879-017-2448-z

**Published:** 2017-05-12

**Authors:** Lander Bosch, Barbara Bosch, Kris De Boeck, Tim Nawrot, Isabelle Meyts, Dominique Vanneste, Cleonice Alexandre Le Bourlegat, Julio Croda, Luiz Vicente Ribeiro Ferreira da Silva Filho

**Affiliations:** 1 Department of Geography, University of Cambridge, Downing Place, Cambridge, CB2 3EN UK; 2 Fundação Oswaldo Cruz Mato Grosso do Sul, Rua Gabriel Abrão s/n, Jardim das Nações, Campo Grande, MS 79081-746 Brazil; 3 St Giles Laboratory of Human Genetics of Infectious Disease, Rockefeller branch, Rockefeller University, 1230 York Avenue, New York, NY 10065 USA; 4 Department of Pediatrics, University Hospitals Leuven, Herestraat 49, 3000 Leuven, Belgium; 5 Centre for Environmental Sciences, Hasselt University, Martelarenlaan 42, 3500 Hasselt, Belgium; 6 Department of Public Health & Primary Care, Occupational & Environmental Medicine, KULeuven, Herestraat 49, 3000 Leuven, Belgium; 7 Division of Geography, KULeuven, Celestijnenlaan 200E, 3001 Leuven, Belgium; 8 Mestrado e Doutorado em Desenvolvimento Local, Universidade Católica Dom Bosco, Av. Tamandaré 6000, Jardim Seminário, Campo Grande, MS 70117-900 Brazil; 9 Fundação Oswaldo Cruz Mato Grosso do Sul, Rua Gabriel Abrão s/n, Jardim das Nações, Campo Grande, MS 79081-746 Brazil; 10 Faculdade de Ciências da Saúde, Universidade Federal de Grande Dourados, Rodovia Dourados, Itahum km 12, Cidade Universitaria, Cx. Postal 533, Dourados, MS 79804-970 Brazil; 11 Instituto da Criança, Hospital das Clínicas, University of São Paulo Medical School, Av. Dr. Enéas Carvalho de Aguiar 647, São Paulo, SP 05403-000 Brazil; 12 Instituto de Ensino e Pesquisa, Hospital Israelita Albert Einstein, Av. Albert Einstein 627, Morumbi, São Paulo, SP 05652-000 Brazil

**Keywords:** Cystic fibrosis, Tuberculosis, Spatial epidemiology, Brazil, Resistance genetics

## Abstract

**Background:**

The reason why Cystic Fibrosis (CF) is the most common fatal genetic disease among Caucasians has been incompletely studied. We aimed at deepening the hypothesis that CF carriers have a relative protection against *Mycobacterium tuberculosis (*Mtb) infection.

**Methods:**

Applying spatial epidemiology, we studied the link between CF carriership rate and tuberculosis (TB) incidence in Brazil. We corrected for 5 potential environmental and 2 immunological confounders in this relation: monthly income, sanitary provisions, literacy rates, racial composition and population density along with AIDS incidence rates and diabetes mellitus type 2. Smoking data were incomplete and not available for analysis.

**Results:**

A significant, negative correlation between CF carriership rate and TB incidence, independent of any of the seven confounders was found.

**Conclusion:**

We provide exploratory support for the hypothesis that carrying a single *CFTR* mutation arms against Mtb infections.

## Background

In Europe, 1:20 to 1:80 people carry a mutation in the Cystic Fibrosis Transmembrane Conductance Regulator (*CFTR*) gene [1], rendering Cystic Fibrosis (CF) the most common life-shortening autosomal recessive disorder among Caucasians. Eighty-seven percent of European patients with CF have at least one F508del allele [2], a deletion of the phenylalanine (F) codon at position 508 [3] and supposed to be a founder mutation in Northern Europe [4].

Thanks to a better understanding and treatment, the mean life expectancy of patients with CF has increased to over 30 years in developed countries nowadays. Until the 1970s however, most CF patients died before reaching reproductive age [5]. However, the reason why CF is still as dominantly present, despite having an expected high purifying index [6], remains unknown.

One hypothesis for this high CF carriership rate among the Caucasian population could be that carrying a single CF mutation has (had) an evolutionary selective advantage. It has been suggested that CF heterozygotes would be more resistant to cholera, typhoid fever or tuberculosis. Using estimates of mortality from TB in different regions, Poolman and Galvani [7] determined that only Tuberculosis (TB) could account for modern-day CF incidence rates in Euro-descendent populations. They suggest that reduced susceptibility to TB in CF heterozygotes could explain the modern gradient of CF in Europe and around the globe, following the *White Plague*.

We aimed at conducting an in-depth analysis of this putative link between CF mutations and TB and decided to do so in Brazil. The country meets four conditions vital to conduct meaningful research on this topic. First, 47•7% of the population are *Brasileiros brancos,* or ‘white Brazilians’ of European descent [8]. This results in significant CF incidence and carriership rate [9]. Second, Brazil is one of the 22 WHO-designated ‘high burden countries’ for TB [10]. Third, the dominant Brazilian *Mycobacterium tuberculosis* (Mtb) strain is the same in Europe and the Americas [11]. Finally, elaborate datasets for CF and TB are available.

Given this unique context in Brazil, we analyse whether support for the CF-TB hypothesis can be provided from a health geographical point of view. This is done through a multidisciplinary, multiscalar spatial epidemiological study, addressing the question: ‘Are Caucasian CF carriership rates in Brazil negatively associated with TB incidence rates when corrected for confounders?’.

## Methods

We researched the link between CF and TB on two scales on the Brazilian territory: the state and municipality level. Demographic data were extracted from the 2010 national population census and 2014 population estimates [12].

The research was approved by the Institutional Ethics Committees and registered through *Plataforma Brasil*.

**Fig. 1:**
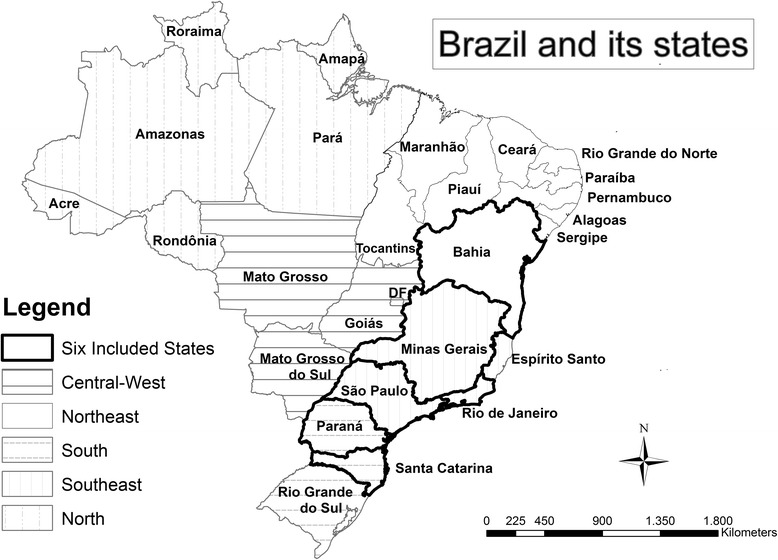
Overview of the 6 Brazilian states included in this research

At the state level, accurate F508del *CFTR* carriership and TB data were available for six states: Bahia (BA), Minas Gerais (MG), Rio de Janeiro (RJ), São Paulo (SP), Paraná (PR) and Santa Catarina (SC) (Fig. 1). Together, these states cover 116 million (m) inhabitants (BA: 15•3 m, MG: 21•0 m, SP: 44•8 m, RJ: 16•7 m, PR: 11•3 m and SC: 6•9 m).

The research of Raskin et al. [13] for MG, SP, PR and SC, Cabello et al. [14] for RJ, and Moura Costa et al. [15] for BA provide the data on F508del carriership. Information on the carriership frequency of other *CFTR* mutations is not systematically reported. TB data per state were obtained from the Health Ministry [16].

As it has been shown that big units of aggregation for TB can lead to associations not being present at smaller [17], a second, more detailed scale was defined: the municipality level. São Paulo state’s 645 municipalities were selected, as well-elaborated CF and TB registries exist for that state: the Brazilian Cystic Fibrosis Registry (REBRAFC) [18] and the national Information System for Notifiable Diseases – TB Registry (SINAN-TB) databases respectively. Eight out of nine main CF Centres of São Paulo State and the SINAN-TB registry approved the use of their anonymised data. For their 907 patients with CF registered in the REBRAFC, the municipality of residence could be identified. SINAN-TB comprises 155,317 registered TB cases in São Paulo state between 2007 and 2014. Again, the patients’ municipality of residence was retrieved.

Minimum CF mutation carriership for the municipalities was calculated multiplying CF prevalence by two, as it is certain both parents of a patient with CF are *CFTR* mutation carriers. The rareness of this disease might cause unrealistically high incidence and carriership rates if, for example, a single CF patient is registered in a relatively sparsely populated municipality. In order to exclude this ‘problem of the small numbers’, only municipalities with over 10,000 inhabitants were withheld.

We hypothesized that the diversity among municipalities would allow correction for several external determinants – potential confounders. Based on a study of the human ecology of both CF and TB, we gathered municipality data on five important environmental confounders: the per capita nominal monthly income, sanitary provisions, literacy rates, the racial composition of the population and the population density [8]. We also studied three comorbid risk factors that are known to impair the immunological response to Mtb: AIDS incidence rates, diabetes mellitus type 2 and smoking [19–21].

Subsequently, a cartographic analysis was performed using GIS software (ArcMap10•2 by ESRI®). Maps were produced for both levels, allowing visual interpretation of the data. Positive spatial autocorrelation might occur when rates of geographically close spatial areas are more likely to be highly related than those from distant areas. This was controlled for, guaranteeing a geographically unbiased analysis at the municipality level.

Next, the municipality level findings were analysed statistically using SPSS (IBM®) and SAS/STAT® software. As data were not normally distributed, we calculated Spearman correlation coefficients between incidence of TB and prevalence of CF (bivariate level). Partial Spearman rank correlations were adjusted for average income, population density, literacy rates, share of Caucasians, sanitary provisions, and AIDS incidence rates (multivariate level). We also applied parametric tests by use of linear regression models instead of Spearman rank correlations to assure consistency in the results. For the parametric tests, the TB incidence data were logarithmically transformed. A *p*-value ≤0•05 was considered statistically significant for all tests.

## Results

Using this multidisciplinary, multiscalar spatial epidemiological approach, we analysed whether Caucasian CF carriership rates in Brazil negatively correlate with TB incidence rates.

**Fig. 2:**
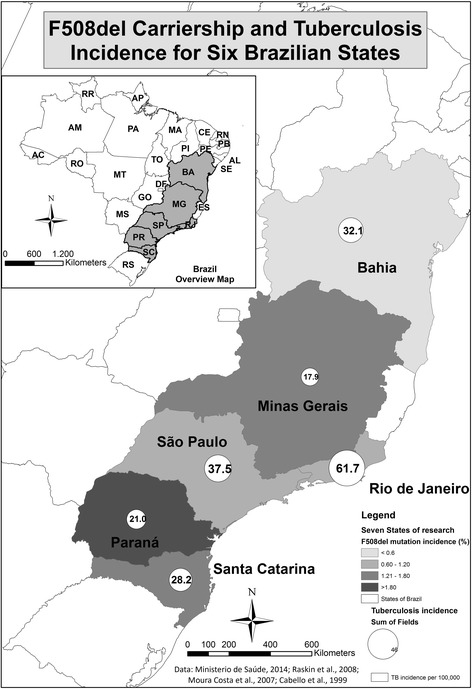
CF carriership prevalence and TB incidence for 6 Brazilian states. In states with a high carriership rate, TB incidence tends to be lower

At the state-level, we analysed F508del carriership rates versus TB incidence (Fig. 2). F508del carriership rates were higher in states with a large share of Caucasians. In the southernmost states for example, PR and SC, on average 80% of inhabitants is considered to be Caucasian [8]. The highest percentages of F508del CF mutation carriers are also noted in those states.

Subsequently plotting F508del carriership against TB incidence, showed a spatial pattern suggesting that states with a higher share of F508del mutation carriers have lower TB incidence rates. PR, SC and MG, while having the highest percentage of carriers (2•38%, 1•79% and 1•37% respectively), are the states with the least TB cases (21•0, 28•2 and 17•9 per 100,000 inhabitants). For RJ, SP and BA, the opposite image is obtained. We conclude to a cartographic trend. Given there are only six observations at state level, carrying out a statistical analysis is not useful.

**Fig. 3:**
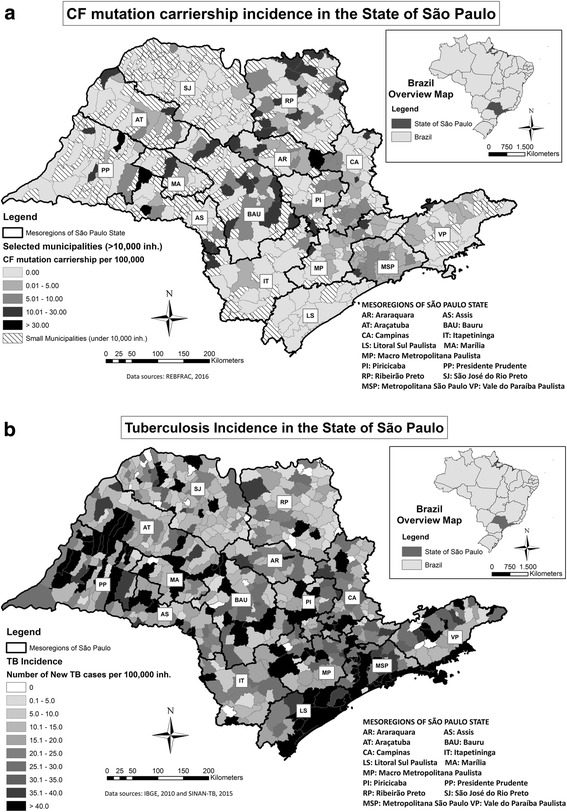
Map of the municipality-level data on the State of São Paulo for CF carriership prevalence (**a**) and TB incidence (**b**)

Figure 3a shows the calculated municipal *CFTR* mutation carriership rates on the municipal level. Figure 3b shows the TB incidence rate for each respective municipality. Again, an inverted pattern between CF mutation carriership and TB incidence rates could be observed.

**Fig. 4:**
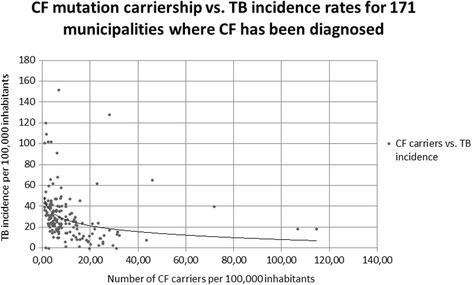
CF carriership versus TB incidence rates for the 171 Sao Paulo municipalities studied

These cartographic observations were complemented by statistical analyses. TB incidence rates were spatially positively autocorrelated (Moran’s Index of 0•08, z-score of 5•44 and *p* < 0•001). A Spearman correlation coefficient (r) was therefore calculated to quantify the CF-TB relationship for the 171 municipalities with over 10,000 inhabitants in which at least one CF case has been registered (Fig. 4). In spite of the probable underestimation of the number of CF carriers in each municipality, CF carriership rate was significantly and inversely correlated with TB incidence (*r* = −0•48 and *p* < 0•001). With adjustments applied for monthly income, population density, literacy rates, the racial composition of the population, sanitary provisions and AIDS incidence rates as partial variables, the corresponding partial Spearman rank correlation was −0•39 (*p* < 0•001). Bivariate analysis of TB or CF with diabetes mellitus type 2 as a confounder was insignificant (*p* = 0.21 and *p* = 0.93 respectively). Data on smoking were not consistently registered and hence unavailable for statistical analysis.

Subsequently, a multivariate analysis was run (Table 1), showing that carriership rates correlate significantly and inversely with log-transformed TB incidence rates. This relation is independent of any of the five potential environmental confounders identified and AIDS incidence rates. Including diabetes mellitus data reduced the power of the overall model strongly so was not used as an explanatory variable.

**Table 1:** Multivariate statistical analysis on the municipality level

Variable [unit]	Estimate	Standard Error	Probability > |t|
Intercept	2•3219	1•2420	0•06
Log (TB incidence rates) [Annual number of new TB cases/100,000 inhabitants]	−0•2091	0•0843	^a^ 0•0141
Average income [Per capita Brazilian Real/Month]	−0•0002	0•0002	0•15
Population density [Inhabitants/km^2^]	−0•0001	0•0000	^b^ < 0•0001
Literacy rates [% of population]	−0•0156	0•0149	0•21
Share of Caucasians [% of population]	0•0050	0•0029	0•08
No sanitary provisions [% of population]	0•0767	0•0355	^a^ 0•0323
AIDS incidence rates [Incidence/100,000 inhabitants]	0•0023	0•0032	0•48

Results of the multivariate analysis of the correlation between CF carriership rates (number of CF carriers per 100,000 inhabitants) and TB incidence (annual number of new TB cases per 100,000 inhabitants) correcting for six potential confounders

^a^indicates significant findings at the 0•05 significance level

^b^indicates significant findings at the 0•001 significance level

## Discussion

This multidisciplinary, multiscalar study in Brazil is, to our knowledge, the first to address the hitherto theoretical link between CF carriership and TB incidence on a contemporary patient dataset. We found a significant, inverse correlation between both, that remained valid after thorough correction for potential environmental and immunological confounders.

Our approach is innovative as it highlights 3 additional aspects to this hypothesis: 1. It extends the idea that human genetics are an important factor in infectious diseases; 2. It studies a possible evolutionary advantage in a representative contemporary environment; 3. It shows how health geography can contribute substantially to elucidating a medical hypothesis.

First, the human genetics of infectious diseases paradigm, the idea that a genetic defect can predispose to a specific infectious pathogen, has revolutionized modern medicine [22]. Increasingly, mutations and new genes are being identified that render the host susceptible to a specific pathogen [23]. Several genetic defects in the defence against TB have been identified [24]. This allows understanding why, whilst most TB-exposed individuals only have latent disease, particular patients develop fulminant TB disease (‘low disease burden’, ‘high susceptibility’). Our research explores the same idea of a genetic determinant in infectious disease. However, it changes the question to why, despite having a high chance of developing disease, some people have relative protection against the disease (‘high disease burden’, ‘low susceptibility’). Important examples of already elaborated ‘resistance genetics’ include sickle cell trait in survival advantage against malaria [25] and the CCR5-delta32 mutation in HIV resistance [26].

Yet, the field of ‘resistance genetics’ is still highly unexplored. Brazil figures among the WHO listed 22 ‘high burden’ countries for TB. At a municipality level, we showed that the group of CF carriers did not have a lower TB burden than other Brazilians [27]. Notwithstanding this high burden, we found an inverse correlation between CF carriership and TB incidence, suggesting a lower susceptibility of CF carriers to TB infection.

Second, in both malaria and HIV, the geographic spread of the ‘resistance allele’ led to the unravelling of the link with its respective infectious disease. Also for our research, the choice of an appropriate place has proven crucial. Brazil is the only country worldwide that combines high TB incidence with the European TB strain and a high CF carriership background, making statistical analyses possible. We were able to study this large cohort thanks to access to the detailed Brazilian registry for TB and CF. In this way evolutionary genetics can be studied in a representative contemporary environment.

Third, this study emphasizes the importance of a multidisciplinary approach to evolutionary genetics. For many years, the question why CF is so frequent amongst Caucasians has been posed in medical literature, yet no in-depth study has been undertaken to more definitively answer this question. We show that Brazil’s geographical, socio-economic and demographic diversity make it possible to approach the role and impact of *CFTR* mutations on TB infection, as well as of various external determinants. This complexity could only be unravelled by means of spatial analysis on medical registry data.

Now, the question goes back to the biomedical bench, as only by cell biological research it will be possible to elucidate and validate the molecular mechanisms behind the found correlation. This biomedical validation is essential to sustain this first preliminary evidence.

The multidisciplinary population-based approach of our study covering 116 million Brazilian inhabitants is a major strength, yet this method also implies several limitations. First, the minimum *CFTR* mutation carriership rates for the municipalities are only a proxy of the real carriership frequency. As registry data were anonymized, it cannot be excluded that siblings are counted as individual patients, introducing an overestimation in the number of carriers. Most probably, however, our data are an underestimation of the CF carrier rate, as siblings with a *CFTR* mutation have not been taken into account, CF carriers not directly related to registered patients could not be identified, and not all CF cases are diagnosed correctly. Brazil has recently initiated systematic newborn screening for CF. As such, many CF cases that would go unnoticed in countries without screening, are now picked up. The frequency of CF disease has been shown to be linked with carriership rate [28].

Next, the structure of both SINAN-TB and REBRAFC registries limited the choice of aerial units that could be chosen for the analyses. The municipality level was the most detailed scale of research possible. Nevertheless, this scale remains susceptible to ecological fallacy. To address this limitation, a case-control study comparing the CF carrier rate of individual Brazilian patients with proven TB to age-, sex- and socio-economically matched healthy individuals from the same municipality could be envisaged. Future research could focus on the genetic background of individual patients with TB.

Lastly, we could not study the link between carrying CF and nontuberculous mycobacterial (NTMs) infection, as systematic municipality data on NTMs are lacking. Primary immune deficiency (PID) patients with Mendelian Susceptibility to Mycobacterial Disease develop severe Mtb and NTM infections [29], and patients with CF have a high susceptibility to infections with NTMs [30]. It can therefore feel counterintuitive that carrying one *CFTR* mutation would not result in an increased but rather a reduced Mtb infection rate. Yet Mtb and NTMs also differ importantly: they constitute a distinct group within the Mycobacteria family and have separate host traits, clinical features [31], and drug resistance patterns [32]. Recent findings in PID support that the immunological response to NTMs might be different from that to Mtb [33].

Notwithstanding these limitations, we found indications for the relative protective role of carrying a single CF mutation against infections with Mtb on two scales on the Brazilian territory.

The possibility that CF carriership has (had) an evolutionary advantage has been raised before. Candidate agents of selective pressure for CF include cholera [34], typhoid fever [35] and tuberculosis. Poolman and Galvani computed that only the European tuberculosis pandemic in the early 1600s can have provided sufficient selective pressure to explain the modern CF incidence [7]. On the other hand, it is also known that a mutation in *CFTR* cannot be fully protective against Mtb infection, as cases of patients with CF and TB have been reported [36].

Never before however, a study has addressed this theoretical hypothesis with patient data.

This observation is relevant, as a rationale for the high CF incidence in Euro-descendants could now be provided. Equally, once a potential human protection mechanism against Mtb infection can be uncovered, this opens up opportunities for the development of new treatment strategies against the disease and implies a vital step towards eradicating TB worldwide.

## Conclusions

Using a multidisciplinary, multiscalar spatial epidemiological approach, we found exploratory support for the hypothesis that carrying a single CF mutation plays a relative protective role against Mtb infections. This could provide a rationale for the continued CF occurrence and encourages biomedical research into the human resistance genetics of tuberculosis and by extension other infectious diseases.

## Abbreviations

CF: Cystic Fibrosis
CFTR: Cystic Fibrosis Transmembrane Conductance Regulator
GIS: Geographical Information System
IBGE: Instituto Brasileiro de Geografia e Estatística
Mtb: *Mycobacterium tuberculosis*
TB: Tuberculosis
WHO: World Health Organization
